# Are novel oral oncolytics underdosed in obese patients?

**DOI:** 10.1007/s00280-023-04601-z

**Published:** 2023-10-31

**Authors:** Lishi Lin, Ellen K. O. van der Meer, Neeltje Steeghs, Jos H. Beijnen, Alwin D. R. Huitema

**Affiliations:** 1 Department of Pharmacy & Pharmacology, The Netherlands Cancer Institute-Antoni Van Leeuwenhoek Hospital, Amsterdam, The Netherlands; 2 Department of Medical Oncology, Netherlands Cancer Institute-Antoni Van Leeuwenhoek, Amsterdam, The Netherlands; 3 Department of Pharmaceutical Sciences, Utrecht University, Utrecht, The Netherlands; 4 Department of Pharmacology, Princess Máxima Center for Pediatric Oncology, Utrecht, The Netherlands; 5 Department of Clinical Pharmacy, University Medical Center Utrecht, Utrecht University, Utrecht, The Netherlands

**Keywords:** Targeted therapy, Obesity, Body weight, Therapeutic drug monitoring, Exposure–response relationship

## Abstract

**Purpose:**

Data on the effects of obesity on drug exposure of oral targeted oncolytics is scarce. Therefore, the aim of this study was to investigate the influence of body weight and body mass index (BMI) on trough levels of oral oncolytics with an exposure–response relationship. The oral oncolytics of interest were abiraterone, alectinib, cabozantinib, crizotinib, imatinib, pazopanib, sunitinib and trametinib.

**Methods:**

This retrospective cohort study included patients treated with the selected oral oncolytics at the standard dose, with a measured trough level at steady state and with available body weight. The Spearman’s correlation test was used to determine the correlation between body weight and trough levels. The Fisher’s exact text was used to compare the frequency of inadequate trough levels between BMI categories.

**Results:**

1265 patients were included across the different oral oncolytics. A negative correlation coefficient was observed between weight and trough levels for crizotinib (n = 75), imatinib (n = 201) and trametinib (n = 310), respectively, ρ = − 0.41, ρ = − 0.24 and ρ = − 0.23, all with a p-value < 0.001. For crizotinib, a higher percentage of patients with a body weight > 100 kg had inadequate trough levels. No statistically significant differences were observed in the frequency of inadequate trough levels between BMI categories.

**Conclusion:**

Higher body weight was only correlated with lower plasma trough levels for crizotinib, imatinib, and trametinib. Therefore, patients with a high body weight may require dose escalation to obtain adequate target levels when treated with these oral oncolytics.

## Introduction

According to the World Health Organization, the global prevalence of obesity has nearly tripled since 1975, with more than 1.9 billion overweight adults and more than 650 million obese adults in 2016 [1]. There is a growing amount of evidence linking obesity to an increased risk of cancer and cancer-related mortality, whereas it can also complicate drug dosing [2].

Currently, oral oncolytics are being administered using fixed dosing strategies for all patients. This also includes obese patients according to the American Society of Clinical Oncology guideline on appropriate systemic dosing in obese cancer patients, as there is not enough evidence indicating the need for a different dosing strategy [3].

However, the fixed, body size-independent dose of oral oncolytics could potentially result in different exposures in obese patients compared to lean patients. Physiological changes that occur in obese patients are for example increased adipose tissue and changed gut and liver enzyme activity. In turn, these physiological changes can alter pharmacokinetic parameters compared to lean patients, such as the oral bioavailability, the volume of distribution and clearance [4]. For example, a drug with high lipophilicity will have affinity for adipose tissue, increasing the volume of distribution and resulting in reduced plasma concentrations. These changes may impact the effectiveness of certain drugs, resulting in different optimal dosing regimens in obese patients.

Literature on the effects of obesity on the exposure of oral oncolytics is still scarce [3]. In a case report, altered pharmacokinetic parameters for sunitinib were described in a morbid obese patient with gastrointestinal stromal tumor. The area under the plasma concentration–time curve of sunitinib in this patient was about 30–50% lower than reported in literature, whereas the steady-state levels of sunitinib were roughly 70% lower than expected [5]. As sunitinib exhibits an exposure–response relationship, lower plasma concentrations could potentially lead to poorer survival outcomes. Therefore, it is of interest to determine whether there is a relationship between obesity and excess weight with plasma concentrations of oral oncolytics with an exposure–response relationship [5–11]. The aim of this study was to investigate the influence of body weight and body mass index (BMI) on trough levels of oral oncolytics with an established exposure–response relationship. The oral oncolytics of interest were abiraterone, alectinib, cabozantinib, crizotinib, imatinib, pazopanib, sunitinib and trametinib. Most of these oral oncolytics exhibit high volumes of distribution, long half-lives and are highly dependent on hepatic clearance [12–19].

## Materials and methods

This retrospective observational cohort study was conducted at the Netherlands Cancer Institute–Antoni van Leeuwenhoek hospital (NKI-AvL), Amsterdam, The Netherlands. Patients treated with the standard oral dose of abiraterone, alectinib, cabozantinib, crizotinib, imatinib, pazopanib, sunitinib and trametinib up until January 2022 with a trough level at steady state were included in this study if there was also a body weight available. Body weight measurements closest to the date of plasma sampling were used, whereas a time window of 30 days before until 30 days after the date of plasma sampling was allowed. A steady state concentration was defined to be reached after five times the half-live of the respective oral oncolytic drug. Two different doses were included for cabozantinib and sunitinib as these different doses are both used frequently. For cabozantinib, 40 mg and 60 mg once daily were allowed, whereas for sunitinib 37,5 mg and 50 mg once daily were allowed. For these two oral oncolytics, linear kinetics were assumed and a 40 mg dose-corrected trough level was determined for cabozantinib and a 50 mg dose-corrected trough level was determined for sunitinib [20, 21]. No other exclusion criteria were applied. The therapeutic target trough levels for abiraterone, alectinib, cabozantinib, crizotinib, imatinib, pazopanib, sunitinib and trametinib were ≥ : 8.4, ≥ 435, ≥ 537, ≥ 235, ≥ 1100, ≥ 20.500, ≥ 50, ≥ 10.6 ng/mL, respectively, as described previously [22, 23]. Trough levels under these therapeutic target trough levels were defined as inadequate trough levels.

At the NKI-AvL, plasma samples of the above mentioned oral oncolytics were collected as part of the standard of care during routine follow-up visits to the outpatient clinic. Plasma samples were measured by validated liquid chromatography with tandem mass spectrometry detection [24–28]. Date and time of the last drug intake and plasma sampling were used to calculate trough levels. Plasma samples collected within half an hour of the last intake were interpreted as trough levels. Log-linear extrapolation was used to determine the trough levels of alectinib, cabozantinib, crizotinib, pazopanib, sunitinib and trametinib, in which their half-lives of 32, 110, 42, 31, 50, and 96 h were used, respectively. For sunitinib, a combined trough level was determined by calculating the sum of sunitinib and the active metabolite N-desethylsunitinib (half-life of 95 h) in plasma samples. Abiraterone trough levels were calculated by multiplying the simulated population trough level by the ratio of the observed plasma concentration versus the population concentration at the corresponding time after dose. This approach was also used for imatinib up to a time after dose of 3 h, whereas log-linear extrapolation was used for a time after dose > 3 h [29].

Patient characteristics were extracted from electronic medical records, whereas data on plasma samples were extracted from the laboratory database. The conduct of this observational cohort study was approved by the Investigational Review Board of the NKI-AvL and, therefore, the need for written informed consent was waived.

Summary statistics included the median and range for continuous variables and frequency and percentages for categorical variables. To determine the correlation between body weight and trough levels_,_ the Spearman’s correlation test was used. Trend lines in the scatterplots were determined using locally estimated scatter plot smoothing. In addition, patients were divided into three groups according to their body weight and BMI. For weight, categories were defined as < 65 kg, 65–100 kg, and > 100 kg. For BMI, categories were defined as lean (< 25 kg/m^2^), overweight (≥ 25 and < 30 kg/m^2^) and obese (≥ 30 kg/m2). The frequency of inadequate trough levels was compared between body weight and BMI categories using Fisher’s exact test. A p-value < 0.05 was considered statistically significant. All statistical analyses were performed in R version 4.2.1 (R Foundation for Statistical Computing, Vienna, Austria).

## Results

A total of 1265 patients were included in this study across the different included oral oncolytics (Table 1). Patients in the abiraterone group had castrate-resistant (n = 295) and hormone-sensitive (n = 13) prostate cancer. All patients in the alectinib (n = 82) and crizotinib (n = 75) groups had non-small-cell lung cancer (NSCLC), whereas all patients in the cabozantinib (n = 49) group had renal cell carcinoma (RCC). One patient in the imatinib group had dermatofibrosarcoma, whereas all other patients in the imatinib group had gastrointestinal stromal tumors (GIST) (n = 200). In the pazopanib group, patients had RCC (n = 45) and sarcomas (n = 75). In the sunitinib group, one patient had a pancreatic neuroendocrine tumor, whereas other patients had GIST (n = 40) and RCC (n = 79). In the trametinib group, patients had melanoma (n = 261) and NSCLC (n = 49). Of the total patient population, 536 (42%) patients were lean, 467 (37%) were overweight and 258 (20%) were obese. BMI could not be calculated for 4 patients due to missing data.

**Table 1:** Patient characteristics for abiraterone, alectinib, cabozantinib, crizotinib, imatinib, pazopanib, sunitinib, and trametinib

	Abiraterone	Alectinib	Cabozantinib	Crizotinib	Imatinib	Pazopanib	Sunitinib	Trametinib
	n = 308	n = 82 (^a^80)	n = 49	n = 75 (^a^74)	n = 201	n = 120 (^a^119)	n = 120	n = 310
Age, median (range)	73 (49–92)	59 (29–88)	60 (33–77)	61 (21–83)	64 (24–88)	57 (18–81)	63 (32–84)	60 (26–93)
Sex, male (%)	308 (100)	42 (51)	37 (76)	37 (49)	125 (62)	59 (49)	92 (77)	162 (52)
ECOG PS (%)								
0	101 (33)	36 (44)	20 (41)	35 (47)	108 (54)	61 (51)	66 (55)	142 (46)
1	143 (46)	36 (44)	16 (33)	36 (48)	35 (17)	50 (42)	30 (25)	91 (29)
2	34 (11)	6 (7)	13 (26)	3 (4)	6 (3)	5 (4)	9 (7)	24 (8)
3	–	1 (1)	–	–	2 (1)	–	–	1 (0.5)
Unknown	30 (10)	3 (4)	–	1 (1)	50 (25)	4 (3)	15 (13)	52 (17)
Height, median (range)	180 (156–204)	175^a^ (150–195)	176 (163–193)	176^a^ (155–198)	175 (150–201)	174^a^ (152–198)	178 (153–197)	175 (145–202)
Weight (%)								
< 65 kg 65–100 kg > 100 kg	7 (2) 235 (76) 66 (22)	13 (16) 62 (76) 7 (8)	2 (4) 43 (88) 4 (8)	17 (23) 53 (71) 5 (6)	26 (13) 154 (77) 21 (10)	18 (15) 84 (70) 18 (15)	6 (5) 101 (84) 13 (11)	63 (20) 207 (67) 40 (13)
BMI group (%)								
Lean (< 25 kg/m^2^)	91 (30)	34 (42)	23 (47)	41 (55)	87 (43)	50 (42)	56 (47)	154 (50)
Overweight (25–30 kg/m^2^)	139 (45)	33 (40)	17 (35)	21 (28)	83 (41)	41 (34)	42 (35)	91 (29)
Obese (≥ 30 kg/m^2^)	78 (25)	13 (16)	9 (18)	12 (16)	31 (15)	28 (23)	22 (18)	65 (21)
Unknown	–	2 (2)	–	1 (1.3)	–	1 (1)	–	–

*ECOG* *PS* Eastern Cooperative Oncology Group Performance Status, *BMI* Body Mass Index ^a^Height of 2 patients in the alectinib group, 1 patient in the crizotinib group and 1 in the pazopanib group were not available.

In the cabozantinib group, 30 patients were treated with 40 mg once daily at time of plasma sampling, whereas the remaining 19 patients were treated with 60 mg once daily. In the sunitinib group, 52 patients were treated with 37,5 mg once daily in a continuous or intermittent schedule, whereas 68 patients were treated with 50 mg once daily in a continuous or intermittent schedule.

**Fig. 1:**
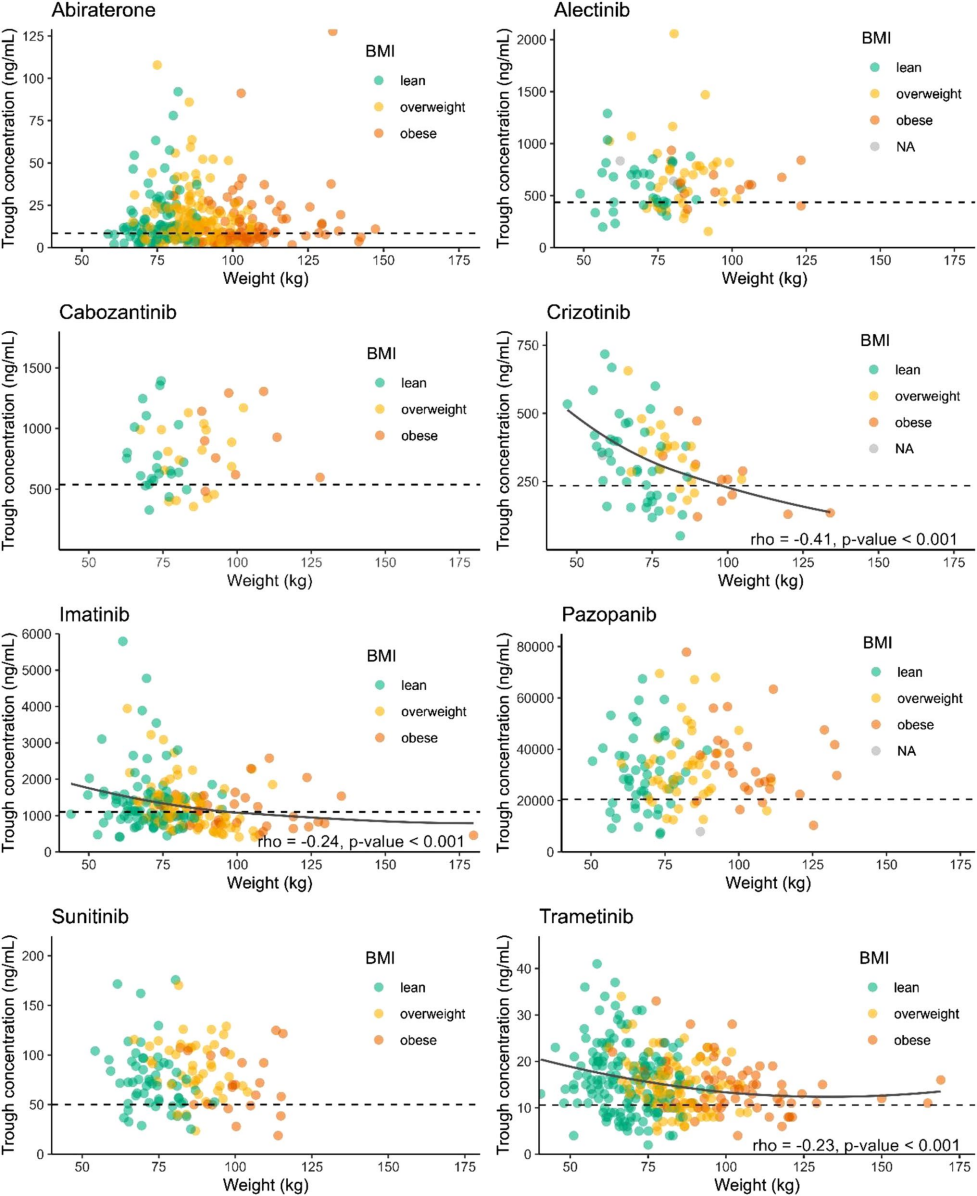
Scatter plots to determine the correlation between weight and trough levels of abiraterone, alectinib, cabozantinib, crizotinib, imatinib, pazopanib, sunitinib, and trametinib. Target trough levels are represented by the dotted horizontal line. BMI, body mass index. NA, not available

Scatterplots of each oral oncolytic are depicted in Fig. 1, in which the correlation between body weight and trough levels was determined for the different oral oncolytics. Statistically significant correlations were found for crizotinib, imatinib and trametinib, with respective correlation coefficients ρ = − 0.41, ρ = − 0.24 and ρ = − 0.23. The trend lines were lower than the target trough level for crizotinib and imatinib at a weight of 100 kg and above. The frequency of inadequate trough levels for each body weight and BMI category of the oral oncolytics are depicted in Tables 2 and 3, respectively. A statistically significant difference was observed between body weight categories for crizotinib and trametinib. For crizotinib, a higher percentage of patients with a body weight > 100 kg had inadequate trough levels, whereas for trametinib, a lower percentage of patients with body weight < 65 kg had inadequate trough levels. For BMI, only a statistically significant difference was observed for sunitinib. In these patients, the frequency of inadequate trough levels was lower in the overweight group, compared to the lean and obese group.

**Table 2:** Frequency of inadequate trough levels within each body weight category for abiraterone, alectinib, cabozantinib, crizotinib, imatinib, pazopanib, sunitinib, and trametinib

	< 65 kg	65–100 kg	> 100 kg	p-value
Abiraterone (n = 308) Inadequate (%)	4/7 (57)	78/235 (33)	25/66 (38)	0.344
Alectinib (n = 82) Inadequate (%)	4/13 (31)	15/62 (24)	1/7 (14)	0.822
Cabozantinib (n = 49) Inadequate (%)	0/2 (0)	8/43 (19)	0/4 (0)	1.000
Crizotinib (n = 75) Inadequate (%)	1/17 (6)	17/53 (32)	3/5 (60)	0.021
Imatinib (n = 201) Inadequate (%)	9/26 (35)	72/154 (47)	13/21 (62)	0.182
Pazopanib (n = 120) Inadequate (%)	5/18 (28)	16/84 (19)	4/18 (22)	0.691
Sunitinib (n = 120) Inadequate (%)	1/6 (17)	26/101 (26)	4/13 (31)	0.909
Trametinib (n = 310) Inadequate (%)	8/63 (13)	59/207 (29)	9/40 (23)	0.031

**Table 3:** Frequency of inadequate trough levels within each BMI categories for abiraterone, alectinib, cabozantinib, crizotinib, imatinib, pazopanib, sunitinib, and trametinib

	Lean	Overweight	Obese	p-value
Abiraterone (n = 308) Inadequate (%)	34/91 (37)	40/139 (29)	33/78 (42)	0.104
Alectinib (n = 80) Inadequate (%)	10/34 (29)	8/33 (24)	2/13 (15)	0.758
Cabozantinib (n = 49) Inadequate (%)	3/23 (13)	4/17 (24)	1/9 (11)	0.596
Crizotinib (n = 74) Inadequate (%)	12/41 (29)	4/21 (19)	5/12 (42)	0.533
Imatinib (n = 201) Inadequate (%)	38/87 (44)	39/83 (47)	17/31 (55)	0.578
Pazopanib (n = 119) Inadequate (%)	13/50 (26)	7/41 (17)	4/28 (14)	0.189
Sunitinib (n = 120) Inadequate (%)	19/56 (34)	4/42 (10)	8/22 (36)	0.008
Trametinib (n = 310) Inadequate (%)	39/154 (25)	23/91 (25)	14/65 (22)	0.841

## Discussion

In this retrospective study, the influence of body weight and BMI on the trough levels of the oral oncolytics, abiraterone, alectinib, cabozantinib, crizotinib, imatinib, pazopanib, sunitinib and trametinib was investigated. A negative correlation was observed between body weight and trough levels for crizotinib, imatinib and trametinib, whereas no significant correlation was observed for the other oral oncolytics. For crizotinib, a higher percentage of patients with a body weight > 100 kg had inadequate trough levels. No statistically significant differences were observed in the frequency of inadequate trough levels between BMI categories. Therefore, body weight seems to be a more important factor than BMI regarding the frequency of inadequate trough levels, as body weight can still vary significantly depending on the severity of obesity. In obese patients, many physiological changes occur and due to the complex interaction of these changes, it remains challenging to predict the impact on pharmacokinetic parameters of a specific drug [4].

In pharmacokinetic analyses of imatinib, higher body weight was associated with higher clearance and apparent volume of distribution, although this was to a small extent [30, 31]. Similarly, for trametinib, higher body weight was associated with higher clearance [32]. These changes may explain the observed negative correlation between body weight and trough levels in our study. In a pharmacokinetic analysis of crizotinib in children, lower clearance and apparent volume of distribution were observed in overweight and obese patients. This led to higher crizotinib exposure in this patient population, which is not in line with our results [33]. In addition, literature on sunitinib in children showed that a high body surface area was associated with higher clearance and volume of distribution, whereas no correlation was observed in our study [34]. It does have to be noted that the last two studies were performed in children, who may exhibit different pharmacokinetic parameters compared to adults.

Exposure to abiraterone, alectinib, cabozantinib, pazopanib and sunitinib and the observed interpatient variability within each group is likely to be influenced to a greater extent by factors other than body weight, as no correlation was observed for these oral oncolytics. For abiraterone and alectinib, food effects probably have a greater influence on plasma levels, whereas for pazopanib, the variable absorption probably plays an important role on the total exposure [35–37].

Apart from the negative correlations found in our study for crizotinib, imatinib and trametinib, the target trough level also influences whether the found correlation between body weight and trough levels is clinically relevant. Although a negative correlation was found for trametinib, the patients with the highest weight still had trough levels above the target trough level. Therefore, negative correlations are only clinically relevant if patients with a high body weight cannot obtain adequate target trough levels. In case of negative correlations in which target trough levels are obtained in patients with a high body weight, it is possible that these patients tolerate oral oncolytics better than lean patients, in case of the presence of an exposure-toxicity relationship [38–40].

One of the strengths of this study is the use of real world data from clinical practice, as plasma samples were collected as part of standard clinical care. However, it should be noted that for some oral oncolytics, only a small number of patients with body weight > 100 kg were included. Therefore, more research is needed to confirm the results of our study. Nevertheless, if a patient with a high body weight appears to have inadequate trough levels, one should be aware of the fact that this may be due to an increased body weight and a dose escalation may be needed to obtain adequate trough levels. One of the limitations of this study is the used method to determine trough concentrations. The estimated trough concentrations of plasma samples collected in the absorption phase are for example underestimated. However, as half-lives are relatively long compared to the dosing interval, the deviations from the actual trough concentrations can be considered acceptable in these cases. Another limitation of our study is the potential of selection bias. We only included patients with a measured plasma level at steady state at the standard dose. Therefore, patients experiencing toxicity leading to dose adjustments in an early phase of the treatment course were excluded. In case the occurrence of toxicity is related to high plasma levels, presumably more low weight patients with high trough levels were excluded, which could have caused an underestimation of the observed negative correlations in our study. For example, in a study with Japanese patients, a low body weight and a small body surface area were correlated with an increased frequency of treatment discontinuation with crizotinib [41].

As it remains challenging to a priori predict the impact of obesity on specific drugs, further research could focus on other novel oncolytic drugs. In addition, the influence of the duration and severity of obesity are important to investigate as these may impact the pharmacokinetics of drugs [42]. Furthermore, obesity is associated with an increased risk of liver failure and hypoalbuminemia [43, 44]. These factors should also be investigated as both can influence the pharmacokinetics of drugs and may act as confounding factors. Lastly, it is of interest to investigate the relationship between plasma concentrations and tissue concentrations in obese patients, as both pharmacokinetic and pharmacodynamic changes can influence the drug effects, whereas the influences of these changes on survival outcomes also needs to be determined.

In conclusion, higher body weight was correlated with lower plasma trough levels for crizotinib, imatinib, and trametinib. Therefore, patients with a high body weight may require dose escalation to obtain adequate target trough levels when treated with these oral oncolytics, which can be monitored using therapeutic drug monitoring. An obesity status alone was not correlated with an increased frequency of inadequate trough levels and no upfront dose adjustment are needed.

## Data Availability

The datasets generated during and analysed during the current study are available from the corresponding author on reasonable request.
